# Galectin-9-Mediated Protection from Allo-Specific T Cells as a Mechanism of Immune Privilege of Corneal Allografts

**DOI:** 10.1371/journal.pone.0063620

**Published:** 2013-05-07

**Authors:** Machiko Shimmura-Tomita, Mingcong Wang, Hiroko Taniguchi, Hisaya Akiba, Hideo Yagita, Junko Hori

**Affiliations:** 1 Department of Ophthalmology, Nippon Medical School, Tokyo, Japan; 2 Department of Ophthalmology, Saitama Medical Center, Jichi Medical University, Saitama, Japan; 3 Department of Ophthalmology, Tokyo Dental College Ichikawa General Hospital, Ichikawa, Chiba, Japan; 4 Department of Immunology, Juntendo University School of Medicine, Tokyo, Japan

## Abstract

The eye is an immune-privileged organ, and corneal transplantation is therefore one of the most successful organ transplantation. The immunosuppressive intraocular microenvironment is known as one of the mechanisms underlying immune privilege in the eye. T-cell immunoglobulin and mucin domain (Tim)-3 is a regulatory molecule for T-cell function, and galectin (Gal)-9 is a Tim-3 ligand. We investigated the role of this pathway in establishing the immune-privileged status of corneal allografts in mice. Gal-9 is constitutively expressed on the corneal epithelium, endothelium and iris-ciliary body in normal mouse eyes and eyes bearing surviving allografts, and Tim-3 was expressed on CD8 T cells infiltrating the allografts. Allograft survival in recipients treated with anti-Tim-3 monoclonal antibody (mAb) or anti-Gal-9 mAb was significantly shorter than that in control recipients. In vitro, destruction of corneal endothelial cells by allo-reactive T cells was enhanced when the cornea was pretreated with anti-Gal-9 mAb. Blockade of Tim-3 or Gal-9 did not abolish anterior chamber-associated immune deviation. We propose that constitutive expression of Gal-9 plays an immunosuppressive role in corneal allografts. Gal-9 expressed on corneal endothelial cells protects them from destruction by allo-reactive T cells within the cornea.

## Introduction

Corneal transplantation is the most successful solid organ transplant in humans [1]–[3]. This clinical experience is matched by the results obtained from experimental models in which orthotopic corneal transplants have been performed in immunocompetent mice and rats. Under these conditions, considerable success has been observed for corneal allografts [4], [5]. The usual explanation for the extraordinary success of orthotopic corneal allografts, either in humans or in experimental animals, is related to the phenomenon of "immune privilege" [4], [6]. In orthotopic grafting, the corneal graft is sutured into an avascular (lacking both blood and lymphatic vessels) rim of recipient cornea. Moreover, the graft necessarily forms the anterior surface of the anterior chamber. The anterior chamber is a well-characterized site of immune privilege where grafts of a variety of foreign tissues are accepted for prolonged and often indefinite intervals [7], [8]. Acceptance of corneal allografts at this site is no exception. Anterior chamber-associated immune deviation (ACAID) is a well-known phenomenon in which antigen (Ag)-specific peripheral tolerance is induced after Ag injection into the anterior chamber [9], [10]. The anterior chamber contains biologically relevant concentrations of various immunomodulatory neuropeptides, growth factors, cytokines, and soluble cell surface receptors, such as alpha-melanocyte-stimulating hormone [11], vasoactive intestinal peptide [12], calcitonin gene-related peptide [13], transforming growth factor (TGF)-β [14], thrombospondin [15], macrophage migration inhibitory factor [16], interleukin (IL)-1 receptor antagonist [17], CD46 [18], CD55 [18], CD59 [18], and CD95L [19]. These factors suppress innate and adaptive immunity and maintain the immunosuppressive microenvironment within the eye [11]–[13], [15]–[19].

Although the site of engraftment is immune privileged, the cornea, when used as an allograft, has also been considered as an immune-privileged tissue. Early experiments by Medawar and by Barker and Billingham indicated that the cornea has the capacity to escape destruction by the alloimmune rejection process [20], [21]. Normal cornea lacks blood and lymphatic vessels [22]. The central part of the cornea, which is used as donor tissue, contains only a small population of major histocompatibility complex (MHC) class II-expressing antigen-presenting cells (APCs) [23]. Although bone marrow-derived cells have recently been reported to be present within normal cornea, most such cells display an immature phenotype lacking MHC class II expression [24]. Moreover, normal corneal cells (i.e., epithelial, stromal, and endothelial cells) express no MHC class II and only weak MHC class I Ags [25]–[27]. In addition, normal corneal endothelial cells (CECs) constitutively express immunomodulatory factors such as CD95L [28], B7-H1 [29] and glucocorticoid-induced tumor necrosis factor receptor family-related protein ligand (GITR-L) [30]. Corneal endothelium is thus considered to play a central role in the protection of corneal allografts from immunological rejection when transplanted orthotopically in the eyes [31] and heterotopically beneath the kidney capsule [32], [33]. The molecular mechanisms of corneal invulnerability are not perfectly understood. Further investigations of the mechanisms underlying immune privilege are necessary to develop new therapeutic approaches to prevent blinding inflammation within the eye, and also the destructive inflammation observed in other tissues and organs.

The T-cell immunoglobulin and mucin domain (Tim) family is a novel group of molecules with a conserved structure and important immunologic functions, including T-cell activation, induction of T-cell apoptosis, T-cell tolerance, and the clearance of apoptotic cells [34]–[36]. Tim-3 is a member of the Tim family specifically expressed on murine T helper (Th)1 cells, but not on Th2 cells [37]. Expression of Tim-3 is detectable only after several rounds of stimulation on CD4 and CD8 cells under Th1 conditions [38], [39]. Tim-3 is also expressed constitutively on macrophages and dendritic cells, and serves opposing roles in the innate and adaptive immune systems [40]. Galectin-9 (Gal-9) has recently been identified as a Tim-3 ligand that negatively regulates Th1 immunity by inducing cell death in effector Th1 cells [41]. Furthermore, Gal-9 can promote Foxp3-positive regulatory T-cell responses and the ligation of Tim-3 with Gal-9 induces apoptosis of effector T cells, but not of regulatory T cells [42]. Although the expression of Tim-3 has been mainly detected in lymphoid tissues, Gal-9 is also expressed in non-lymphoid organs such as the liver, small intestine, kidney, lung, and cardiac muscle [43], [44]. Gal-9, similar to other galectins, modulates a variety of biological functions such as cell aggregation and adhesion, apoptosis of tumor cells, and others [44], [45].

More recently, Gal-9 has been reported to ameliorate a representative autoimmune model, collagen-induced arthritis, by inducing the apoptosis of synoviocytes, suppressing the generation of Th17 cells, and up-regulating the induction of regulatory T cells [46], [47]. These results suggest that the Tim-3/Gal-9 pathway induces immunotolerance in animals with various immune responses. In the eye, Tim-3 and Gal-9 interaction is reported to suppress herpes simplex virus-specific CD8 T cell immunity. [42], [48] However, to the best of our knowledge, the roles of this interaction in immune privilege have yet to be explored.

The present study demonstrates that Gal-9 is constitutively expressed in ocular tissue. We used blocking Abs to Tim-3 and Gal-9 to investigate whether those molecules play any role in establishing the immune-privileged status of corneal allografts. Our data indicate that the interaction between Tim-3 and Gal-9 plays an important role in the survival of corneal allografts. Constitutive expression of Gal-9 on CECs plays a role in protecting CECs from destruction by allo-reactive T cells.

## Materials and Methods

### Ethics statement

This study was carried out in strict accordance with the recommendations in the Guide for the Care and Use of Laboratory Animals of the National Institutes of Health. All experiments conducted in this study were carried out under Nippon Medical School Institutional Animal Care and Use Committee approved protocol number H23-81.

### Mice and anesthesia

Male BALB/c, C57BL/6, and C3H/He mice were purchased from Sankyo Lab Service. All mice were used at 8–10 weeks old and were treated according to the Association for Research in Vision and Ophthalmology guidelines on the use of animals in research. The protocol for this animal study was reviewed and approved by our institutional review committee. Each mouse was anesthetized by intramuscular injection of a mixture of 3.75 mg of ketamine and 0.75 mg of xylazine before all surgical procedures.

### Abs and flow cytometry

Anti-mouse Tim-3 mAb (RMT3-23, rat IgG2a), and anti-mouse Gal-9 mAbs (RG9-1, rat IgG2b, and RG9-35, rat IgG2a) were generated as previously described [49], [50]. For fluorescence immunohistochemistry or flow cytometry, mAbs against CD4 (GK1.5, rat IgG2b), CD8 (53.6.72, rat IgG2a), CD11b (M1/70), CD11c (N418, hamster IgG), and Gal-9 (RG9-35, rat IgG2a) were used. All FITC-, PE-, allophycocyanin-, or biotin-conjugated mAbs and isotype control Ig were obtained from eBioscience. Biotin-conjugated anti-Tim-3 antibody was obtained from R&D systems. Culture supernatant from the 2.4G2 hybridoma (anti-CD16/CD32 mAb) was used to block non-specific binding, and 2.4G2 was obtained from ATCC. For detecting apoptosis, Annexin V apoptosis detection kit FITC (eBioscience) was used according to the instructions from the manufacturer. Stained cells were then analyzed (FACSCant 2 and BD FACSDiva software; BD Biosciences).

### Orthotopic corneal transplantation and treatment

Penetrating keratoplasty was performed as previously described [29]. Briefly, 2-mm-diameter donor corneas were placed in the same sized recipient bed with eight interrupted sutures (11-0 nylon; Mani). Sutures were removed at 8 days post-grafting. C57BL/6 mice were used as donors and BALB/c mice were used as recipients. Three times a week for 8 weeks, 0.2 mg of anti-mouse Tim-3 mAb (RMT3-23), anti-mouse Gal-9 mAb (RG9-1 or RG9-35), or control rat IgG was administered intraperitoneally.

### Evaluation of corneal allograft

Orthotopic grafts were observed by operative microscopy at least twice a week. A masked assessment of orthotopic corneal grafts was performed by a single observer (M.W.), who examined each graft for survival according to a previously reported scoring system that defines graft survival as follows: 0, clear graft; 1+, minimal superficial nonstromal opacity; 2+, minimal deep stromal opacity with pupil margin and iris vessels visible; 3+, moderate deep stromal opacity with only the pupil margin visible; 4+, intense deep stromal opacity with the anterior chamber visible; and 5+, maximum stromal opacity with total obscuration of the anterior chamber [29]. Grafts with opacity scores of 2+ or greater after 3 weeks were considered to have been rejected.

### RT-PCR

Cornea, iris-ciliary body, and neural retina were isolated from a total of 10 normal mouse eyes. Total RNA was extracted from each tissue using STAT 60 (TEL-TEST). First-strand cDNA was prepared using a SuperScript First Strand Synthesis System (Invitrogen Life Technologies) from 5 µg of total RNA. Standardization of cDNA samples was based on the content of β-actin cDNA. Primers for mouse β-actin were 5′-ACAATGAGCTGCGTGTGGCC-3′ and 5′-ACGGCCAGGTCATCACTATTG-3′. Primers for mouse Tim-3 were 5′-CTGCAGGATACAGTTCCCTG-3′ and 5′-ACGTCAACAGCCAGCAGC-3′. Primers for mouse Gal-9 were 5′-ACTTTCAGAACAGCTTCAATGGA-3′ and 5′-AGTCCATCATGATATCAGGCAAT-3′. PCR was performed in a total volume of 20 µl in PCR buffer in the presence of 0.2 mM dNTP, 1 µM of each primer, and 1 U of *Taq* DNA polymerase (Advanced Biotechnologies). After 32 cycles of amplification, PCR products were separated by electrophoresis on a 2% agarose gel and visualized by ethidium bromide staining.

### Histology and immunohistochemistry

Eyes bearing corneal allografts were removed for histological assessment at 2 and 4 weeks after transplantation. For immunohistochemistry, normal and graft-bearing eyes were removed and frozen in Optimal Cutting Temperature compound (Sakura Finetechnical) in acetone-dry ice and stored at −80°C. Cryostat sections (5 µm) were fixed in cold acetone, followed by immunofluorescent staining for the detection of mouse Tim-3, Gal-9, CD4, CD8, CD11c, and CD11b. Briefly, after blocking with 2% BSA, sections were incubated with FITC-, PE-, or biotin-conjugated primary Ab diluted to 4 µg/ml for 2 h. This was followed by staining with FITC-conjugated anti-rat IgG (eBioscience) or streptavidin-FITC (Jackson ImmunoResearch Laboratories) that was diluted to 4 µg/ml for 1 h at room temperature. After washing with PBS, sections were mounted with PI-containing or 4,6-diamidino-2-phenylindole (DAPI)-containing mounting medium and observed under confocal microscopy (LSM710; Zeiss).

### In vitro assay of corneal endothelial cell destruction by alloreactive T cells

To examine corneal endothelial destruction by alloreactive T cells in vitro, we constructed a model of the efferent phase of corneal rejection in culture dishes [29]. Fresh normal corneas from C57BL/6 eyes were incubated with 10 µg of anti-Gal-9 mAb (RG9-35) or control rat IgG for 2 h in 5% CO_2_ at 37°C, and then washed three times with PBS. T cells were purified from the spleens of BALB/c mice that had been presensitized by subcutaneous immunization with C57BL/6 spleen cells or with third-party (C3H/He) spleen cells, or from the spleens of naive BALB/c, C57BL/6, or C3H/He mice, using the MACS magnetic cell sorting and separation system (Miltenyi Biotec) with a Pan T cell Isolation kit (anti-CD45R/B220, anti-CD49b/DX5, anti-CD11b/Mac-1, and anti-Ter119 mAbs; Miltenyi Biotec), according to the instructions from the manufacturer. Purified T cells (96–98% pure as estimated by FACSCalibur; BD Biosciences) were suspended in RPMI 1640. Corneas pretreated with anti-Gal-9 mAb or control rat IgG were incubated with 2.5×10^5^ T cells for 6 h in 5% CO_2_ at 37°C, then washed three times with PBS. Unfixed corneal samples were incubated with 50 µg/ml of propidium iodide (PI) for 30 min to stain the nuclei of dead endothelial cells. Using confocal microscopy (×40 magnification), PI-positive cells were counted in three randomly selected areas in the corneal endothelium of each corneal sample, as previously described [29]. As a positive control for corneal cell death, normal C57BL/6 cornea was incubated with Triton X-100 without Ab treatment or incubation with T cells. As negative controls, normal C57BL/6 corneas with Ab treatment and incubation without T cells, and corneas without Ab treatment or incubation with T cells were used.

### Assessment of donor-specific ACAID

ACAID induction was tested as previously described [29]. Briefly, recipient BALB/c mice received an anterior chamber (AC) injection of 5×10^5^ donor C57BL/6 spleen cells. One week after AC injection, recipients were immunized by subcutaneous injection of 1×10^7^ C57BL/6 spleen cells. Seven days after immunization, 1×10^6^ irradiated (2000 rad) C57BL/6 spleen cells were injected into the right ear pinnae. Twenty-four hours after the ear challenge, ear thickness was measured using a low-pressure micrometer (Mitsutoyo; MTI). Ear swelling was determined as follows: specific ear swelling  =  (measurement of right ear at 24 h-measurement of right ear at 0 h)-(measurement of left ear at 24 h-measurement of left ear at 0 h)×10^−3^ mm. Ear swelling responses at 24 h after injection are presented as individual values (×10^−3^ mm) for each tested animal and as a group mean ± standard error of the mean. For 3 weeks, treatments with anti-Tim-3, anti-Gal-9 mAb, or control rat IgG mAb were performed starting from the day of AC injection and continuing until the day of ear injection. As a positive control, a similar number of irradiated spleen cells were injected into the right ear pinnae of BALB/c mice that had been immunized 1 week previously by subcutaneous injection of 1×10^7^ C57BL/6 spleen cells. As a negative control, 1×10^6^ irradiated C57BL/6 spleen cells were injected into the right ear pinnae of naïve mice that had not been previously AC injected or immunized.

### Statistical analyses

Corneal graft survival rates were compared using Kaplan-Meier survival curves and the Breslow-Gehan Wilcoxon test. Ear-swelling measurements, corneal endothelial cell death, infiltrating CD4 positive and CD8 positive cells, and percentage of gated spleen and LN cells were analyzed using the two-tailed Student's t test. Probability (p) values <0.05 were considered statistically significant.

## Results

### Expression of Gal-9 and Tim-3 in normal mouse eyes

**Figure 1:**
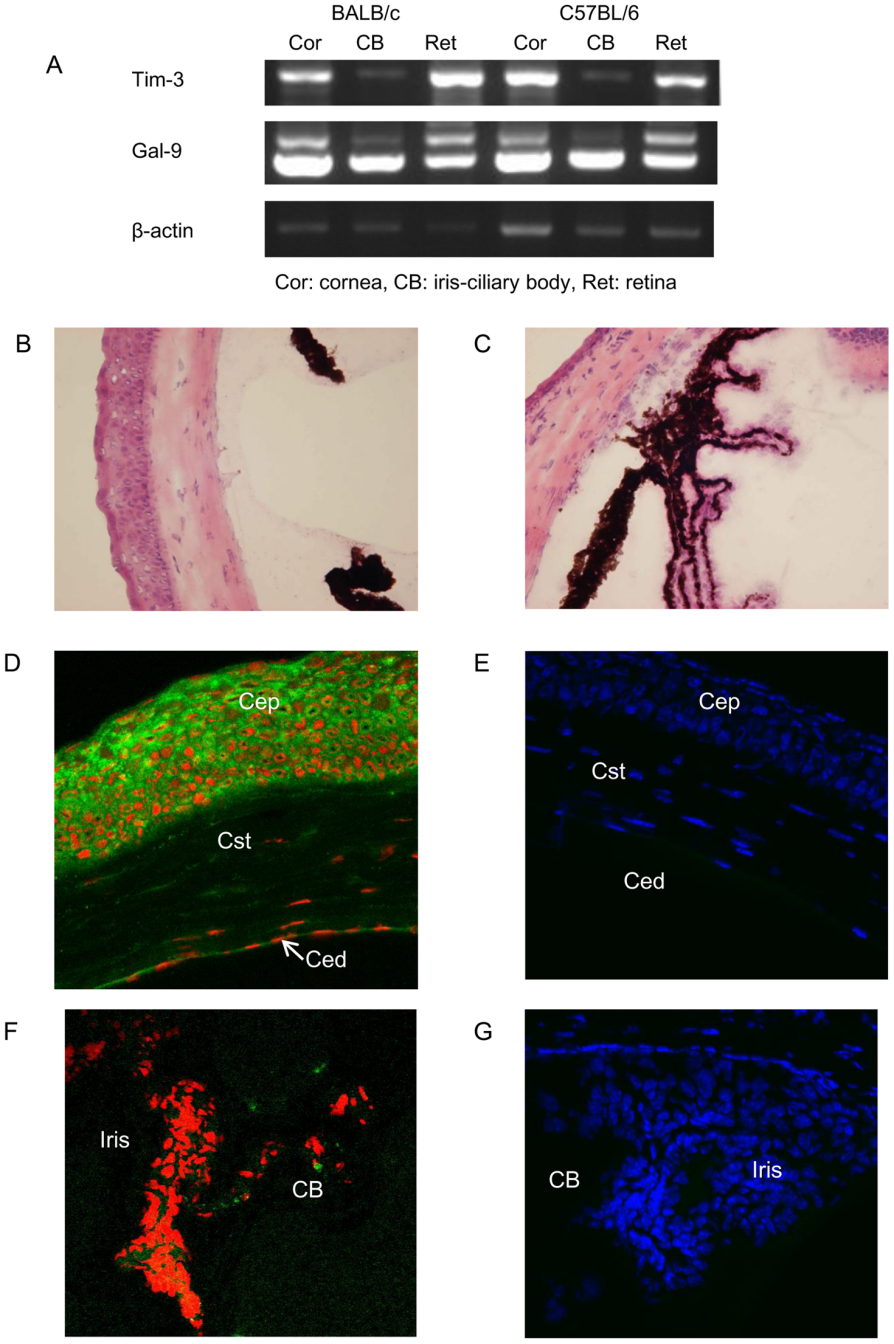
Expression of Tim-3 and Gal-9 in normal eye tissue. (**A**) mRNA was extracted from freshly isolated cornea, iris-ciliary body, and neural retina of normal mouse eyes, and then reverse transcribed and amplified by PCR. PCR products were electrophoresed in 2% agarose gel and visualized by ethidium bromide staining. (**B**) Section of normal mouse cornea was stained using hematoxylin and eosin (HE). (**C**) Section of normal mouse iris-ciliary body was stained using HE. (**D and F**) Cryostat sections were stained with anti-Gal-9 mAb followed by FITC-conjugated anti-rat IgG (green). Nuclei were stained with PI (red). (**E and G**) Cryostat sections were stained with biotinylated anti-Tim-3 Ab followed by streptavidin-FITC (green). Nuclei were stained with DAPI (blue). Ced, corneal endothelium; Cst, corneal stroma; and Cep, corneal epithelium. Original magnification, ×40.

Gal-9 mRNA was strongly expressed in freshly isolated cornea, iris-ciliary body, and the neural retina according to RT-PCR (Fig. 1A). Normal mouse cornea and iris-ciliary body with HE staining were showed in Fig. 1B, C. Immunofluorescent staining indicated that the expression of Gal-9 was localized to corneal epithelium and endothelial cells, but was absent in the corneal stroma (Fig.1D). Gal-9 protein was also expressed in the iris-ciliary body of normal mouse eyes (Fig.1F). RT-PCR revealed that Tim-3 mRNA was expressed in freshly isolated cornea and neural retina, and weakly expressed in the iris-ciliary body of normal mouse eyes (Fig. 1A). However, the expression of Tim-3 protein was not found by immunofluorescent staining in normal cornea or the iris-ciliary body (Fig.1E, G).

### Blockade of Gal-9 or Tim-3 accelerates corneal allograft rejection

**Figure 2:**
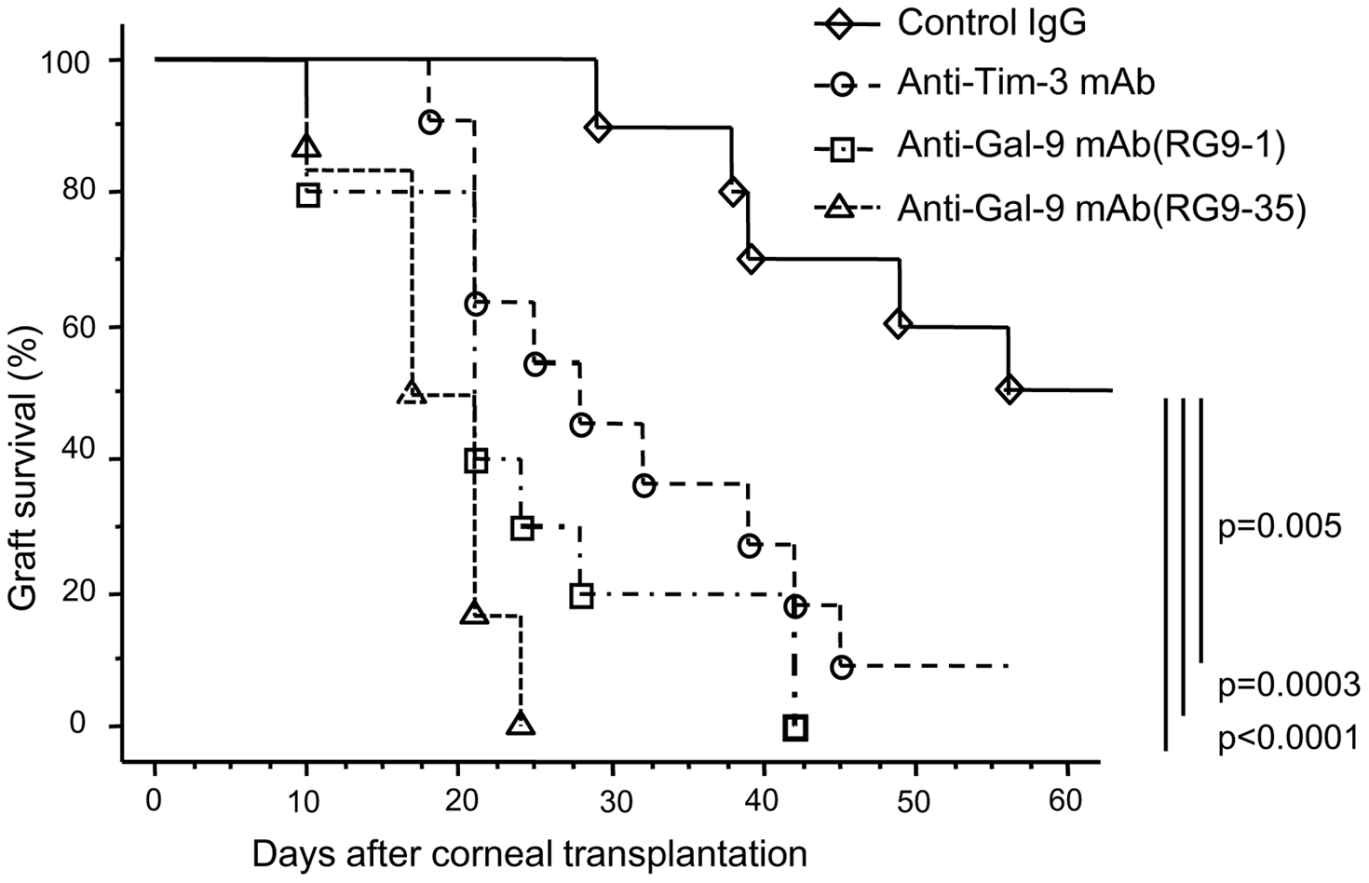
Blockade of Tim-3 or Gal-9 accelerates corneal allograft rejection. Normal corneas of C57BL/6 mice were transplanted orthotopically into normal eyes of BALB/c mice. After the grafting procedure, recipients were intraperitoneally injected with 0.2 mg of anti-Tim-3 mAb, anti-Gal-9 mAb (RG9-1 or RG9-35), or control rat IgG three times a week for 8 weeks. Graft survival was clinically assessed and compared. Survival of allografts treated with anti-Tim-3 or anti-Gal-9 mAb was significantly shorter than that in control allografts (*n* = 9–10 in each group).

Normal corneas of C57BL/6 mice were transplanted orthotopically into normal eyes of BALB/c mice. In all recipients, 0.2 mg of anti-Tim-3 monoclonal antibody (mAb), anti-Gal-9 mAb, or control rat IgG was administered intraperitoneally 3 times a week for 8 weeks after grafting. Graft survival was clinically assessed and compared. Approximately 50% of allografts survived >8 weeks in the control IgG-treated recipients (Fig. 2). We have previously reported that approximately 50% of corneal allografts from C57BL/6 donors survive in untreated BALB/c recipients [29]. Therefore, administration of the control IgG did not affect corneal allograft survival. In contrast, all allografts were rejected within 50 days when recipients were treated with anti-Gal-9 mAbs, and 90% allografts were rejected after treatment with anti-Tim-3 mAb. Survival of allografts in anti-Tim-3 or anti-Gal-9 mAb-treated mice was significantly shorter than that in control IgG-treated mice (anti-Tim-3, p = 0.005; anti-Gal-9(RG9-1), p = 0.0003; anti-Gal-9(RG9-35), p<0.0001) (Fig. 2).

### Expression of Gal-9 and Tim-3 in surviving corneal allografts and secondary lymphoid organs

**Figure 3:**
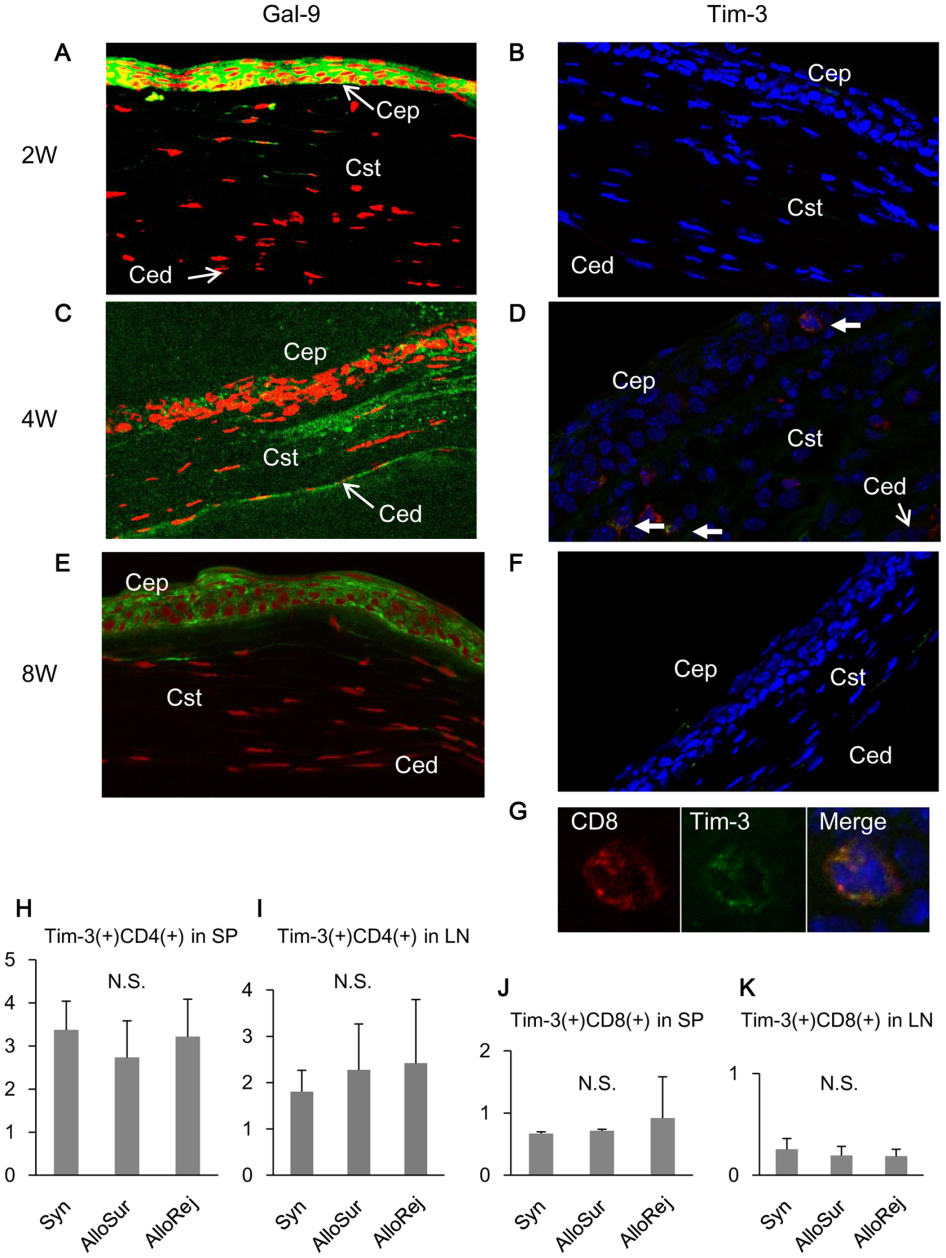
Expression of Tim-3 and Gal-9 in corneal allografts and secondary lymphoid organs. Normal corneas of C57BL/6 mice were transplanted into normal eyes of BALB/c mice. Graft-bearing eyes were isolated at 2, 4, and 8 weeks. (**A, C, and E**) Cryostat sections of surviving graft centers were examined by immunofluorescent staining with anti-Gal-9 mAb followed by FITC-conjugated anti-rat IgG mAb or streptavidin-FITC (green). Nuclei were stained with PI (red). (**B, D, F, and G**) Cryostat sections of surviving graft centers were examined by immunofluorescent staining with biotinylated anti-Tim-3 Ab followed by streptavidin-FITC (green), and PE-conjugated anti-CD8 mAb (red). Nuclei were stained with DAPI (blue). Arrows in D show the Tim-3+ CD8+ cells, and G represents the magnification of one of the double positive cells. Cep, corneal epithelium; Cst, corneal stroma; Ced, corneal endothelium. Original magnification, ×40. (**H, I, J, and K**) Whole spleen (SP) or LN cells from recipients bearing surviving (Allo Sur) or rejected (Allo Rej) allografts and syngeneic (Syn) grafts were analyzed by flow cytometry. Data are displayed as the proportion of Tim-3+ CD4+ cells in the whole SP and LN (H, I), Tim-3+ CD8+ cells in the whole SP and LN (J, K). Data are presented as the mean ± standard deviation of five recipients in each group. N.S., not significant.

When normal corneas of C57BL/6 were transplanted orthotopically into normal eyes of BALB/c mice, Gal-9 was strongly expressed on corneal epithelium, endothelial cells, and superficial stromal cells in the surviving corneal allografts (Fig. 3A, C, E). Tim-3-expressing cells were present in corneal epithelium and stroma at the center of the surviving allograft at 4 weeks after transplantation (Fig. 3D). To identify the character of these Tim-3 positive cells, we performed double-staining with CD4, CD8, CD11b, and CD11c. These cells were only CD8 positive (Fig. 3G). Tim-3 positive cells were not present in short or long term surviving corneal allografts (Fig. 3B, F). Proportions of Tim-3 expressing CD4+ or CD8+ T cells in splenocytes and lymph node (LN) cells were statistically indistinguishable among recipients bearing surviving allografts, those bearing rejected allografts, and those bearing syngeneic allografts (Fig. 3H, I, J, K), indicating that expression of Tim-3 in the spleen and lymph nodes does not correlate with the fate of corneal grafts.

### Blockade of Gal-9 or Tim-3 increases T cell infiltrations in allografts

**Figure 4:**
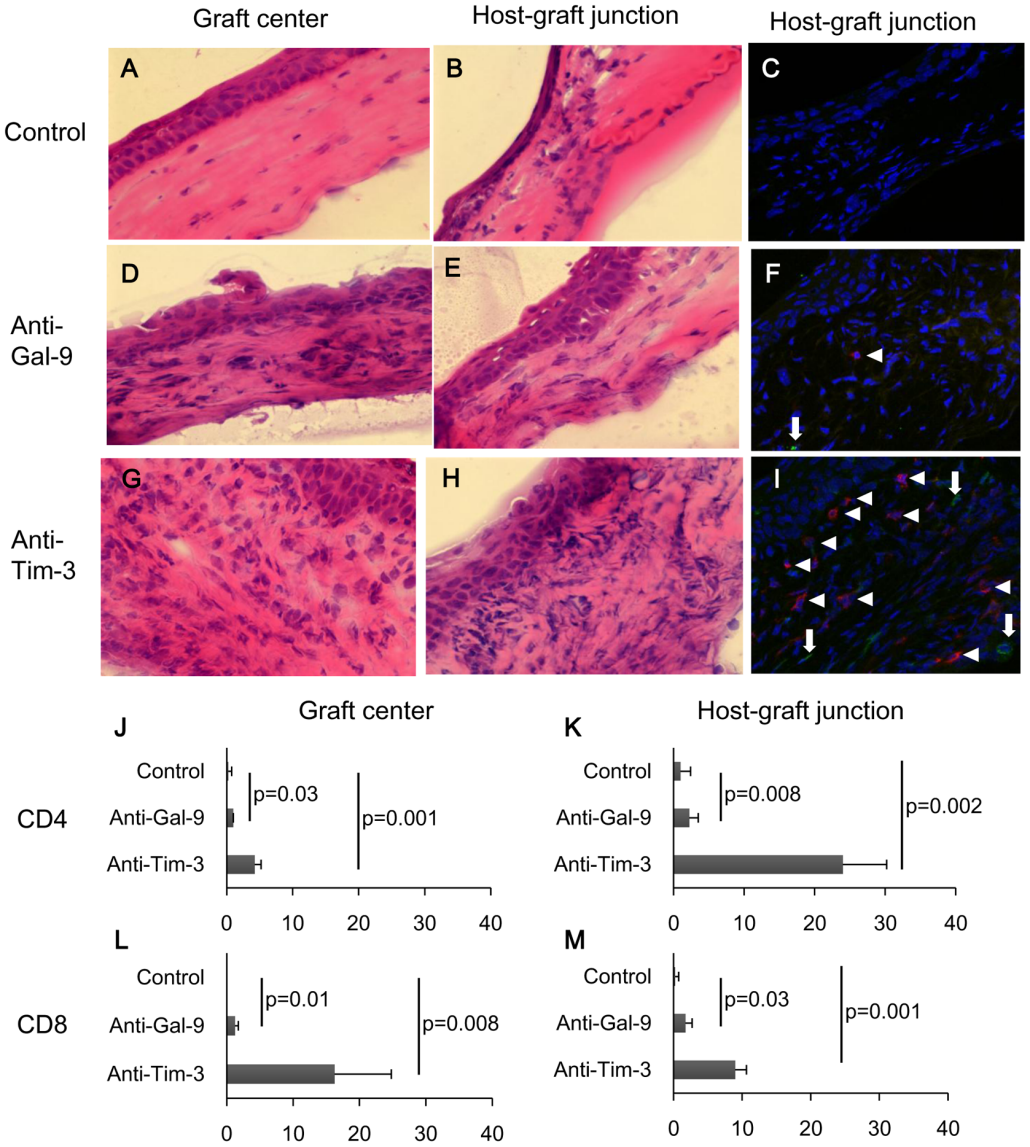
Blockade of Gal-9 or Tim-3 increases CD4 and CD8 T cell infiltration in allografts. Normal corneas of C57BL/6 mice were transplanted into normal eyes of BALB/c mice. All recipients were treated with anti-Tim-3 mAb, anti-Gal-9 mAb, or control rat IgG. Graft-bearing eyes were isolated at 4 weeks. (**A, B, D, E, G, and H**) Cryostat sections of graft-bearing eyes were examined by HE staining. (**C, F, and I**) Cryostat sections of graft-bearing eyes were examined by immunofluorescent staining with PE-conjugated anti-CD4 mAb (red) and FITC-conjugated anti-CD8 mAb (green). Nuclei were stained with DAPI (blue). Arrow heads in F and I show CD4+ cells and arrows in F and I show CD8+ cells. Original magnification, ×40. (**J, K, L, and M**) The number of CD4+ and CD8+ cells at graft center and host-graft junction in recipients treated with anti-Tim-3 mAb and anti-Gal-9 mAb was greater as compared to control recipients. Data are presented as the mean ± standard deviation of four corneas in each group.

We next examined the graft-bearing eyes from anti-Gal-9 or anti-Tim-3 treated recipients at 4 weeks. HE staining showed that the number of infiltrating cells was increased by the anti-Gal-9 or anti-Tim-3 treatment at the graft center (Fig. 4A, D, G) as well as the host-graft junction (Fig. 4B, E, H). Immunofluorescent staining revealed that infiltration of both CD4+ T cells and CD8+ T cells was increased by the anti-Gal-9 or anti-Tim-3 treatment (Fig. 4C, F, I). The numbers of infiltrating CD4+ T cells and CD8+ T cells at graft center and host-graft junction were significantly increased in anti-Gal-9 or anti-Tim-3 treated recipients compared to control (Fig. 4J, K, L, M).

### Gal-9 protects corneal endothelial cells from killing by alloreactive T cells in vitro

**Figure 5:**
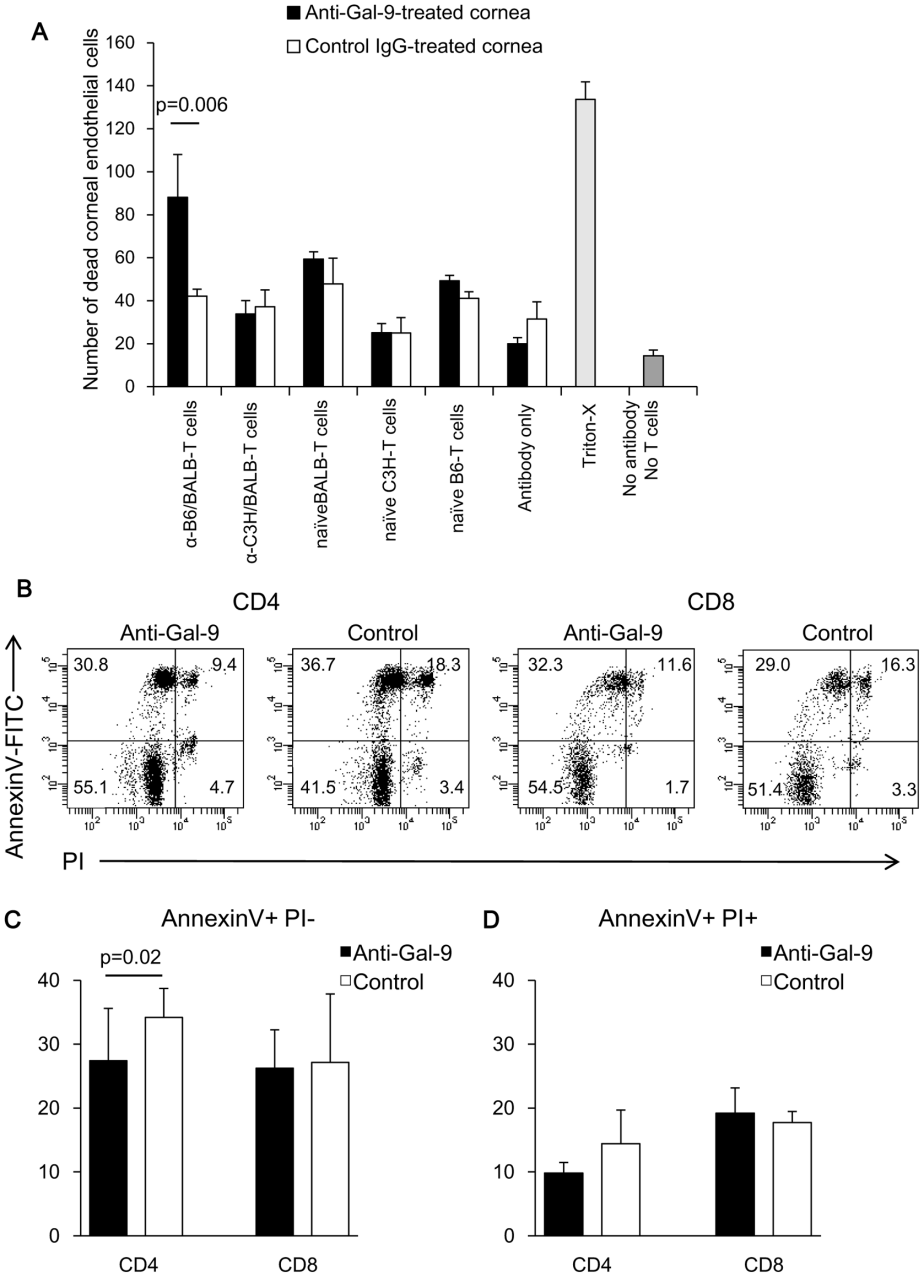
Gal-9 protects corneal endothelial cells from killing by alloreactive T cells. (**A**) C57BL/6 (B6) corneas pretreated with anti-Gal-9 mAb (RG9-35) or control IgG were incubated with purified T cells from the spleens of BALB/c mice presensitized against B6 alloantigens (α-B6/BALB-T cells) or third-party C3H/He alloantigens (α-C3H/BALB-T cells), or from the spleens of naïve BALB/c (naïve BALB-T cells), B6 (naïve B6-T cells), or C3H/He mice (naïve C3H-T cells). After a 6-h incubation, corneal endothelial cell death was detected by staining unfixed tissue with PI followed by confocal microscopic examination. Positive control corneas were incubated with Triton X-100, without Ab treatment or incubation with T cells. As negative controls, corneas with Ab treatment and incubation without T cells (antibody only), and corneas without Ab treatment or incubation with T cells (no antibody no T cells) were used. Data are presented as the mean ± standard error of five corneas in each group. (**B, C**) Apoptosis of allo-reactive T cells following co-incubation with B6 corneas pre-treated with anti-Gal-9 mAb or control IgG was examined by flow cytometry with FITC-conjugated Annexin V/PI. Apoptosis of CD4+ T cells was significantly suppressed with anti-Gal-9 mAb treatment compared to control, while there was no difference in apoptosis of CD8+ T cells. B shows the representative flow cytometry data. C shows the data presented as the mean ± standard deviation of six experiments in each group.

The constitutive Gal-9 expression in surviving corneal allografts led us to postulate a hypothesis, in which the Gal-9 molecules have the capacity to protect corneal allografts from alloreactive infiltrating T cells by inducing apoptosis of these effector cells. To address this possibility, we used a model of corneal endothelial cell destruction by alloreactive T cells in vitro that we had established previously [29]. In previous studies by us and others, corneal endothelial cells have been documented to represent a target of alloreactive T cells in both human and rodent corneal transplantations [51]. As a model of the effector phase of corneal rejection, normal C57BL/6 corneas were incubated with purified T cells from the spleens of BALB/c mice presensitized against C57BL/6 alloantigens. Purified T cells from the spleens of BALB/c mice presensitized against third-party C3H/He alloantigens were used as non specifically activated T cells. Splenic T cells from naive BALB/c, C57BL/6, and C3H/He mice were used as allogeneic, syngeneic, and third-party naive T cells, respectively. The number of dead CECs was significantly increased in anti-Gal-9 mAb-treated corneas than in control IgG-treated corneas after incubation with allo-reactive T cells (*p* = 0.0006) (Fig. 5A). In contrast, no significant difference was observed between anti-Gal-9 mAb- and control IgG-treated corneas when activated T cells against third-party allo-antigens, naïve BALB/c or C3H/He T cells, and syngeneic C57BL/6 T cells were used as the effector cells. These results suggest that Gal-9 protects corneal endothelial cells from killing by allo-reactive T cells. When the co-culture of allo-reactive T cells and corneal endothelial cells were treated with anti-Gal-9 mAb, apoptosis of CD4+ T cells was significantly suppressed compared to control, while there was no difference in apoptosis of CD8+ T cells (Fig. 5B, C, D).

### Blockade of Tim-3 or Gal-9 does not abolish ACAID

**Figure 6:**
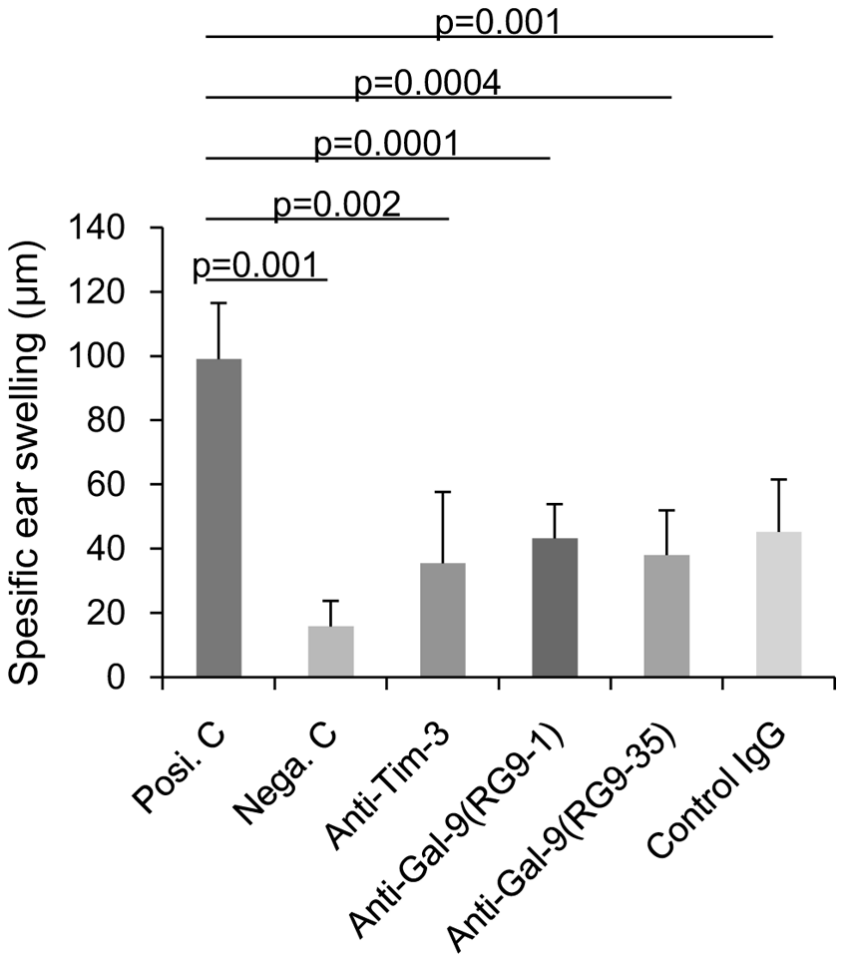
Blockade of Tim-3 or Gal-9 does not abolish ACAID. C57BL/6 spleen cells were used as alloantigens and injected into the right AC of BALB/c normal eyes. Two weeks later, C57BL/6 spleen cells were injected subcutaneously to sensitize the mice. After one more week, a challenge was conducted by injecting C57BL/6 spleen cells into the right ear pinnae of each mouse, and specific ear swelling was measured 24 h later as an indication of DH. Treatments with anti-Tim-3 mAb, anti-Gal-9 mAb (RG9-1 or RG9-35), or control rat IgG were performed starting from the day of AC injection and continuing until the day of ear challenge. DH responses were similarly suppressed in anti-Tim-3, anti-Gal-9, and control IgG-treated groups, with no significant differences observed among them. Positive control mice (Posi.C) received subcutaneous immunization and ear challenge without previous AC injection. Negative control mice (Nega.C) received only the ear challenge without AC injection or immunization. Data are presented as the mean ± standard error of five corneas in each group.

Eye-associated tolerance, termed ACAID, is one of the major mechanisms underlying the immune privilege of the eye and maintains acceptance of corneal allografts [10]. We hypothesized that the vulnerability of corneal allografts noted after blockade of Tim-3/Gal-9 might result from a failure to induce ACAID. We therefore tested the effect of Tim-3/Gal-9 blockade on alloantigen-specific ACAID induction using a simple model. B6 spleen cells were used as alloantigens and injected into the right anterior chamber of normal BALB/c eyes. Two weeks later, B6 spleen cells were injected subcutaneously to sensitize the mice. After one more week, B6 spleen cells were introduced into the ear pinnae to determine the delayed hypersensitivity (DH) response 24 h later. For 3 weeks, treatments with anti-Tim-3 or anti-Gal-9 mAb were applied starting from the day of anterior chamber (AC) injection and continued until the day of ear challenge. The DH response was induced in sensitized mice without prior AC injection (positive controls) as compared with unsensitized naïve mice (negative controls) (Fig. 6). Prior AC injection significantly suppressed the DH response in control IgG-treated mice, indicating the induction of ACAID. Treatments with either anti-Tim-3 or anti-Gal-9 mAb did not significantly affect the induction of ACAID (Fig. 6). These results indicate that Tim-3/Gal-9 interaction is not involved in the induction of ACAID.

## Discussion

The present study has investigated whether Tim-3 and its ligand Gal-9 are involved in the immune-privileged status of the eye using the corneal allo-transplantation model and explored potential underlying mechanisms.

We demonstrated for the first time that normal eye tissue, such as cornea, iris-ciliary body, and neural retina constitutively express Gal-9. Gal-9 was localized on corneal epithelium and endothelial cells in normal cornea. Tim-3 expression was detected at the mRNA level by RT-PCR, but was undetectable at the protein level by immunohistochemistry in normal eye tissue, suggesting a post-transcriptional regulation. This may also be explained by the fact that PCR is much more sensitive in detecting low levels of transcript compared to immunohistochemistry for protein expression.

Expression of Gal-9 in corneal cells was retained after corneal allo-transplantation, and Tim-3-expressing CD8+ T cells were present in grafts 4 weeks after transplantation but disappeared after 8 weeks after transplantation. Tim-3 has been known as a marker for exhausted effector T cells, and interaction with Gal-9 leads to selective death of Tim-3+ cells[52]. Therefore, our results might suggest that Gal-9 expressed in the corneal tissue eluminated Tim-3+CD8+ T cells. If Tim-3 or Gal-9 were blocked by injection of neutralizing mAbs in recipients of corneal allografts, the allografts became more vulnerable to rejection and had more infiltration of both CD4+ and CD8+ T cells. These results indicate that the Tim-3/Gal-9 interaction plays an important role in protecting corneal allografts from immune rejection. Constitutive expression of Gal-9 on corneal epithelium and endothelial cells is thus at least partially responsible for the immune-privileged status of corneal allografts.

Tim3+CD8+ T cells observed in surviving allograft epithelium and superficial stroma may help grafts to survive by maintaining tolerance in the eye. It has been established that Tim-3 and PD-1 together work in inducing T cell exhaustion and tolerance in skin transplantation model and acute myelogenous leukemia model [53], [54]. Therefore, it is also possible that Tim-3 and PD-1 coexpression is involved in the immune privilege observed in corneal transplantation. However, while we found that PD-1 was expressed in CD4 T cells attached to the corneal endothelium of allografts in our previous report [29], Tim-3 was only expressed in CD8+ T cells infiltrating the epithelium and stroma of allografts in this report. Therefore, PD-1 might not be expressed in these Tim-3+ CD8+ T cells. Furthermore, their respective ligands B7-H1/B7-DC and Gal-9 also show different expression patterns. We have already shown that anti-PD-1 show similar effects to anti-Tim-3 [29], suggesting that PD-1 and Tim-3 may have complementary roles in targeting alloreactive CD4 and CD8 T cells. Further investigation is needed to clarify this point.

We explored two possible mechanisms for the Gal-9-mediated protection of corneal allografts from rejection. One possibility was that Tim-3/Gal-9 interaction might be involved in the induction of Ag-specific systemic immune tolerance to eye-derived Ags, known as ACAID. Ags placed in the anterior chamber are captured by resident APCs, which then migrate through the trabecular meshwork out of the eye and into the blood. Once these cells reach the marginal zone of the spleen, active TGF-β, IL-10, and CCL5 attract and activate Ag-specific CD4 and CD8 T cells, which differentiate into Ag-specific regulatory T cells that inhibit induction and expression of delayed hypersensitivity [10]. Induction of donor-specific ACAID has been confirmed to be associated with long-term graft acceptance and promotes the survival of corneal allografts [10]. Our results demonstrate that ACAID was induced in recipients treated with control IgG, because Ag-specific DH was suppressed. DH was also similarly suppressed in recipients treated with anti-Tim-3 or anti-Gal-9 mAb. These results indicate that the induction of ACAID is independent of the Tim-3/Gal-9 interaction and that the acceptance of corneal grafts can be abrogated even if ACAID remains intact. Therefore, the Gal-9-mediated protection of corneal allografts from immune rejection is due to a mechanism other than ACAID.

Another possible mechanism is corneal Gal-9-mediated local protection of corneal allografts from effector T cells. Therefore, we evaluated corneal endothelial cell destruction by alloreactive T cells *in vitro*. Interestingly, the killing of corneal endothelial cells by alloreactive T cells in vitro was significantly enhanced in corneas pretreated with anti-Gal-9 mAb, as compared to those pretreated with control IgG. This finding demonstrates that Gal-9 expressed on CECs plays a substantial role in the protection of corneal endothelium from destruction by alloreactive effector T cells. In contrast, no significant differences were observed between anti-Gal-9 mAb- and control IgG-treated corneas with naïveC57BL/6, BALB/c or C3H/He T cells, and activated T cells against third-party allo-antigens. Apoptosis of alloreactive CD4 T cells co-cultured with corneas was significantly inhibited by anti-Gal-9 mAb. This suggests that Gal-9 locally protects the corneal endothelium by inducing apoptosis of alloreactive CD4 T cells. This can also explain the fact that treatment with anti-Tim-3 and anti-Gal-9 mAbs enhanced infiltration of CD4 T cells in the host-graft junction. On the other hand, anti-Gal-9 treatment had no effect on the apoptosis of alloreactive CD8 T cells. Taken together with the fact that anti-Tim-3 treatment enhanced the infiltration of CD8 T cells in the graft center more than anti-Gal-9 treatment, some Tim-3 ligand other than Gal-9 may be involved in the inhibition of allograft rejection by CD8 T cells.

The underlying mechanisms for Gal-9-mediated protection of corneal endothelial cells from alloreactive T cells within the cornea are still unclear. Gal-9 is not only leading to selective deletion of Tim-3+ cells, but also it induces Foxp3+Tim3+ regulatory T cells, in other organ transplantation models[52]. In the herpes simplex virus infection models, Gal-9/Tim-3 interaction involved direct inhibitory effects on Tim-3+CD8+ effector T cells as well as the promotion of Foxp3+ regulatory T cell activity[48]. Gal-9 has been also reported to suppress T cell proliferation and infiltration through a Tim-3-independent mechanism. Gal-9 prevents CD40 induced proliferative responses of CD40+ effector T cells[55]. Further studies are needed to address these possible mechanisms for Gal-9-mediated corneal local protection from alloreactive T cells. In summary, the present findings indicate that the Tim-3/Gal-9 pathway plays a critical role in maintaining the immune-privileged status of corneal allografts. Gal-9 is constitutively expressed on corneal endothelium and protects against destruction by alloreactive effector T cells within the cornea. The cornea thus exists as an immune-privileged tissue in part due to Gal-9-mediated protection.
